# Geographically-stratified HIV-1 group M *pol* subtype and circulating recombinant form sequences

**DOI:** 10.1038/sdata.2018.148

**Published:** 2018-07-31

**Authors:** Soo-Yon Rhee, Robert W. Shafer

**Affiliations:** 1 Division of Infectious Diseases, Department of Medicine, Stanford University, Stanford, CA 94301, USA

**Keywords:** Genetic variation, HIV infections, Retrovirus, Viral epidemiology

## Abstract

Accurate classification of HIV-1 group M lineages, henceforth referred to as subtyping, is essential for understanding global HIV-1 molecular epidemiology. Because most HIV-1 sequencing is done for genotypic resistance testing *pol* gene, we sought to develop a set of geographically-stratified *pol* sequences that represent HIV-1 group M sequence diversity. Representative *pol* sequences differ from representative complete genome sequences because not all CRFs have *pol* recombination points and because complete genome sequences may not faithfully reflect HIV-1 *pol* diversity. We developed a software pipeline that compiled 6,034 one-per-person complete HIV-1 *pol* sequences annotated by country and year belonging to 11 pure subtypes and 70 CRFs and selected a set of sequences whose average distance to the remaining sequences is minimized for each subtype/CRF and country to generate a Geographically-Stratified set of 716 *Pol* Subtype/CRF (GSPS) reference sequences. We provide extensive data on *pol* diversity within each subtype/CRF and country combination. The GSPS reference set will also be useful for HIV-1 *pol* subtyping.

## Background & Summary

Accurate classification of HIV-1 group M lineages, henceforth referred to as subtyping, has been essential for understanding the evolution of divergent HIV-1 in the context of the global pandemic. The classification of such sequences is complicated by the HIV-1’s high mutation rate and propensity to develop new recombinant forms when two different virus strains infect the same cell. Indeed, HIV-1 group M sequences can be classified into many different lineages referred to as pure subtypes and circulating recombinant forms (CRFs).

The HIV research community led by researchers at the Los Alamos National Laboratories (LANL) HIV Sequence Database established criteria for the taxonomic recognition of the pure subtypes A, B, C, D, F, G, H, J, and K and for an increasing number of CRFs^1,2^. A new CRF has generally been established and assigned a number when a recombinant virus with unique breakpoints has been sequenced in its entirety and identified in three or more epidemiologically unlinked individuals. Recombinant viruses that do not share breakpoints with an established CRF are classified as unique recombinant forms (URFs)^3,4^.

Several online programs use phylogenetic or bioinformatic approaches to determine the subtype/CRF of a submitted sequence. Each of these programs relies on a set of ~9,700 bp full-genome reference sequences for each subtype and for a large proportion of CRFs^5–9^. The vast majority of HIV-1 sequencing, however, is done for genotypic resistance testing and is therefore confined to the *pol* gene (2,841 bps; 947 amino acids) which encodes the protease, reverse transcriptase (RT), and integrase enzymes. We therefore sought to develop a set of representative *pol* sequences for subtyping of HIV-1 *pol* sequences. Selecting representative *pol* sequences for each subtype/CRF based solely on *pol* will differ from selecting them based on complete genome sequences because not all CRFs have breakpoints in *pol*. Furthermore, sequences selected based on their complete genome may not reflect the diversity of HIV-1 *pol* because there are fewer complete genome sequences than *pol* sequences.

As many subtypes and CRFs exhibit regional divergence^10–13^, we developed a systematic approach for selecting a Geographically-Stratified *Pol* Subtype/CRF (GSPS) reference dataset. For each distinct subtype/CRF and country combination, we characterized the extent of diversity in the *pol* gene and applied a partitioning around medoids (PAM) algorithm to identify the smallest number of centrally located sequences that would minimize the average distance to the closest leaf (ADCL) of the complete set of subtype/CRF/country sequences^14^. This approach is designed to select a subset of sequences that is designed to represent the diversity of a larger collection of sequences^14^.

It should be noted, however, that the collection of published HIV-1 sequences may not perfectly represent the entirety of global HIV-1 sequences. Indeed, as we indicate in this study, many CRFs are likely over-represented in public sequence databases compared with their prevalence in HIV-1 infected persons. To counter this fact, we supplemented our GSPS reference dataset with a set of additional sequences that were closest to the progenitors of many of the reported CRFs.

**Figure 1:**
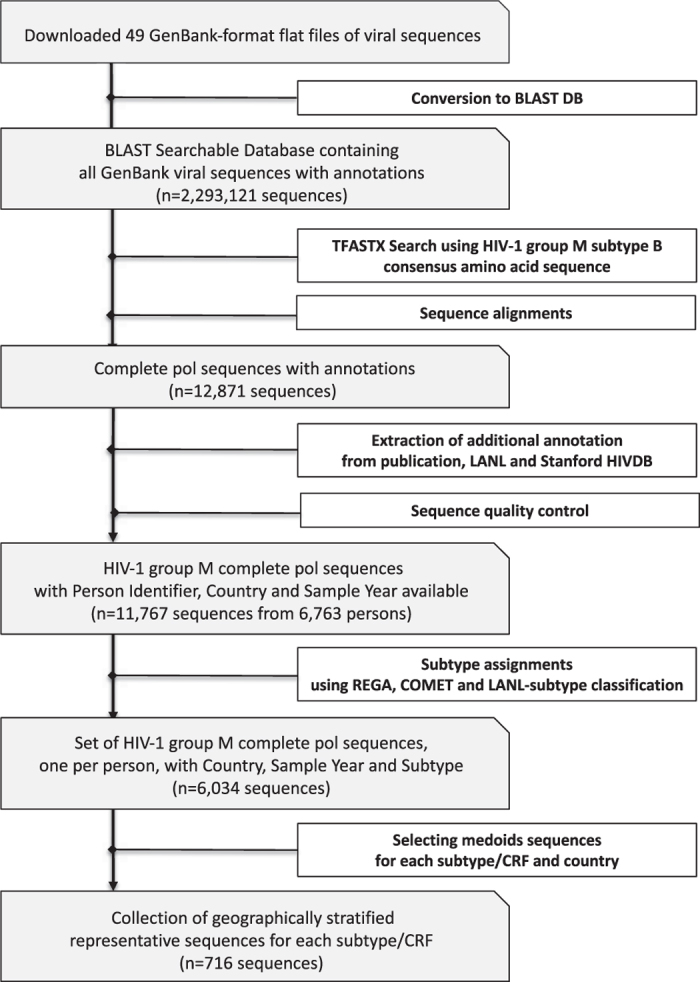
Schematic overview of the process of creating a set of Geographically-Stratified *Pol* Subtype/CRF (GSPS) reference sequences.

Figure 1 shows the complete process of (1) Identifying complete *pol* sequences from GenBank; (2) Extracting annotation from GenBank, LANL^1^, the HIV Drug Resistance Database^15^, and associated PubMed publications; (3) Performing sequence quality control; (4) Assigning a consensus subtype to those *pol* sequences for which there was an agreement using two or more of the following subtyping classifications: LANL^1^, Rega subtyping tool^5^, and COMET^6^; and (5) Selecting the sequences that comprise the GSPS reference set. In addition, the manuscript also characterizes the extent of *pol* diversity within each subtype/CRF and country combination and demonstrates the potential usefulness of the GSPS set of sequences for subtyping studies.

## Methods

### Compilation of HIV-1 group M complete *pol* sequences

We accessed the repository of NCBI-GenBank Flat File Release 220.0 (release date of June 15, 2017)^16^ and downloaded all files containing GenBank virus sequence records (gbvrl1.seq.gz – gbvrl49.seq.gz). Using a custom Perl script (GB_to_Fasta.pl), we parsed each of the 2,293,121 virus sequence records and extracted data from the following GenBank fields: “TITLE”, “AUTHORS”, “TaxonID”, and, if available, “PUBMED”, “collection_date”, “country”, and “notes”. We created an fasta sequence file in which each sequence header contained the extracted sequence annotations. We then converted the fasta sequence file to a BLAST-searchable database using MAKEBLASTDB (NCBI blast+ package)^17^.

To retrieve sequences homologous to the HIV-1 subtype B *pol* consensus amino acid sequence, we searched the database using TFASTX (FASTA package)^18^. TFASTX is a local sequence alignment program which searches translated nucleotide sequences using an amino acid query sequence. Unlike TBLASTN (NCBI blast+ package), it can extend an alignment across reading frames^19^. Using an e-value of 0.0000001, TFASTX returned 54,781 High-scoring Segment Pairs (HSPs). We then selected the 12,871 HSPs with a similarity score >80 and 947 amino acids. To remove frameshifts and insertions and to generate a nucleotide sequence alignment of 2,841 bps in length, we used the global nucleotide sequence alignment program, GGSEARCH (FASTA package). A second custom Perl script (Gene_to_Sequences.pl) was developed to streamline the above processes.

The 12,871 HIV-1 complete *pol* sequences were grouped into 631 submission sets containing the same submission “Title” and “Authors”. Approximately 85% of the 631 submission sets were linked to one or more publications containing a PubMed ID. To describe the provenance of each sequence (i.e., whether it was the only sequence obtained from an individual vs. whether it was one of multiple sequences obtained from an individual), we created a Person ID descriptor for each sequence using annotations extracted from the GenBank record, LANL, HIVDB, and published papers.

From the set of 12,871 complete HIV-1 *pol* sequences, we excluded 1,104 sequences including 55 non-group M sequences and 1,049 sequences that either lacked annotation for country or sample year (n=339), lacked a Person ID descriptor (n=234), or had poor sequence quality (n=476) defined as the absence of ≥10 amino acid positions or the presence of ≥3 stop codons, ≥20 highly unusual mutations, or ≥5 signature APOBEC-associated mutations^20^. These exclusions left us with 11,767 well-characterized *pol* sequences from 6,763 persons. One sequence per person was then selected for subtyping. If one complete *pol* sequence was available from a person, we selected the complete *pol* sequence from that person. Otherwise, we used the random number generator implemented in mysql with a seed and chose one random sequence from that person.

### Subtype assignment

For the 6,763 one-per-person complete *pol* sequences, we recorded the subtype classification in the LANL database, which is based on the author-defined subtype and additional analyses performed by LANL. This classification is based on the entire sequence in a GenBank record and is therefore influenced by sequence outside of the *pol* region. We also submitted each of the *pol* sequences to the online subtyping programs Rega and COMET^5,6^. Sequences for which two or more of three classifications agreed were assigned a consensus subtype/CRF.

Overall, 6,034 (89.2%) of the 6,763 sequences were assigned a consensus subtype/CRF: 11 pure subtypes including A2 and F2 (65.6% of 6,034) and 70 CRFs (34.4% of 6,034). The sequences assigned a subtype included (i) 5,091 sequences (84.4% of 6,034) for which the classifications assigned by Rega, COMET, and LANL yielded the same classification; (ii) 875 (14.5% of 6,034) for which two of the three approaches yielded the same classifications; and (iii) 68 (1.1% of 6,034) for recently recognized CRFs in LANL but for which reference sequences did not exist in Rega and COMET.

Thirty-nine sequences assigned to seven CRFs were reclassified because they did not have recombination breakpoints in *pol*: CRF14_BG, CRF15_01B, CRF32_06A1, CRF41_CD, CRF46_BF1, CRF57_BC and CRF88_BC were reclassified based on their *pol* sequence to subtype G, CRF01_AE, CRF06_cpx, subtype D, subtype F1, subtype C, and subtype C, respectively. Twenty sequences assigned to CRF70_BF1, CRF71_BF1 and CRF72_CRF1 were not included because the breakpoints in their *pol* genes were inconsistent^21^. The majority of the remaining 709 sequences (10.5% of 6,763), for which the three approaches diverged, were URFs typically reported as unassigned by Rega and COMET. They were excluded from subsequent analyses because URFs are not recognized established lineages. Most of these URFs contained sequence segments consistent with CRF01 and B (Southeast Asia), B and C (South East Asia), B and F (South America), and A and D (East Africa).

The overall distribution of subtypes in the 6,763 *pol* sequences in order of frequency was as follows: subtype B (32.2%), subtype C (18.0%), CRF01_AE (17.9%), Other CRFs (11.2%), URFs (10.5%), subtype A (4.6%), CRF02_AG (1.9%), subtype D (1.3%), and subtype G (1.1%). The remaining four uncommon subtypes F, H, J, and K comprised 1.4% of the dataset. Table 1 compares this distribution to the estimated global distribution of subtypes from a 2011 publication describing the WHO/UNAIDS Network characterizing the global and regional distribution of subtypes^22^. This comparison shows that our dataset of complete *pol* sequences was more likely to contain viruses belonging to subtype B, CRF01_AE, other CRFs, and URFs and less likely contain viruses belonging to subtypes C, A, and CRF02_AG compared those in the WHO/UNAIDS publication.

The 6,034 one-per-person, subtype-assigned *pol* sequences were from 88 countries in the following seven regions: Asia (43.5%), Sub-Saharan Africa (26.4%), North America (12.9%), Europe (8.2%), Latin America and Caribbean (7.2%), former Soviet Union (1.5%) and North Africa and Middle East (0.4%) (Complete.Set.txt, Data Citation 1). The countries with the greatest number of subtype/CRFs were China (18), Cameroon (16), DRC (15), Spain (12) and Brazil (11). Twenty-nine countries had 3 to 9 subtype/CRFs and 55 countries had 1 to 2 subtype/CRFs. The number of subtype/CRFs per country was influenced in large part by the frequency with which laboratories in a country performed complete *pol* sequencing.

The subtypes present in the largest number of countries were B (42 countries), C (30 countries), A1 (24 countries), CRF02_AG (20 countries), CRF01_AE (15 countries), D (13 countries), G (13 countries), F1 (10 countries) and CRF06_cpx (10 countries). Sequences from more than 100 persons were available for five subtype/CRFs including B (n=2,175), C (n=1,215), CRF01_AE (n=1,210), A1 (n=306), CRF07_BC (n=300) and CRF02_AG (n=130). The median sampling year was 2007 (IQR: 2003-2009). Fifty-four percent of the sequences were complete genome sequences.

### Creating a set of Geographically-Stratified *Pol* Subtype (GSPS) reference sequences

To identify a set of representative sequences, we applied the k-medoids method using the partitioning around medoids (PAM) algorithm implemented in the Reference Package PReparare (rppr) program in the pplacer package^14^. Medoids are representative members of a dataset whose average distance from all the members in the dataset is minimized. The rppr program takes a phylogenetic tree and the number (k) of medoids (leaves or sequences) to be subsampled that minimize the average distance to the closest leaf (ADCL). We performed this analysis for each set of three or more sequences from the same country within a subtype/CRF.

Overall, there were 294 distinct combinations of subtypes/CRFs and country (subtype/CRF/country) of which 155 contained three or more sequences. For these 155 sequence sets, we created a neighbour-joining tree rooted at its midpoint using the TN93 substitution model. We then used rppr to identify the set of sequences that minimized ADCL for each k with k ranging from 1 to the number of sequences in the tree (n)–1.

After plotting, k vs. ADCL, for each subtype/CRF/country combination (ADCL.txt, Data Citation 1), we decided to select the minimum set of k sequences that lowered ADCL below 4.0% because this could be achieved for 72% (111) of the 155 subtype/CRF/country combinations. However, for 14 (9%) subtype/CRF/country combinations for which there was much sequence diversity and for which the k required to lower ADCL below 4.0% was high, we selected the set of k sequences that lowered ADCL below a higher threshold: (i) 4.5% for subtype A1 in Kenya, Tanzania, and Uganda; for subtype B in Cyprus, Germany, Denmark, Spain; for subtype C in Botswana, Ethiopia, Kenya, Tanzania, and South Africa; and for subtype G in Nigeria; and (ii) 5.0% for subtype B in Brazil. For the remaining 30 subtype/CRF/country combinations (19%) for which the ADCL remained above 4.5% regardless of k, we included all 141 sequences.

For 139 subtype/CRF/country combinations with just one or two sequences, we included just one sequence unless the two sequences belonging to that subtype/CRF/country differed by more than 4.0%.

Overall the above procedures yielded 684 sequences including (i) 523 sequences from the 155 subtype/CRF/country combinations containing three or more sequences and (ii) 161 sequences from the 139 subtype/CRF/country combinations containing just one or two sequences.

### Supplementing GSPS sequences with subtype sequences closest to CRF progenitors

The set of publicly available HIV-1 sequences, particularly sequences contributed by research laboratories, is likely to contain a higher relative proportion of CRF than non-CRF sequences compared with their prevalence in HIV-1 infected patients because CRF sequences may be more likely to be published. If a set of reference sequences contains a CRF sequence but does not contain the non-CRF ancestors that gave rise to the CRF, some non-recombinant sequences may appear to be more similar to the CRF reference sequence than to the parent subtype. This is particularly likely to occur when the CRF is dominated by one subtype and contains just a small segment of another subtype.

To counter this effect, we added 32 additional reference sequences from subtypes A1, A2, B, C, D, F1, G, and CRF01_AE using the following approach. We used the LANL CRF gene maps to identify 49 CRFs comprising one or two of the following subtype/CRFs: A1, A2, B, C, D, F1, G, and CRF01_AE in their *pol* genes. We then created 60 neighbour-joining trees containing each of the sequences belonging to 49 CRFs and each of the sequences from that CRF’s dominant *pol* subtype according to the CRF gene map. Each of these trees comprised just the region of *pol* shared by the CRF and its parent subtype. For several CRFs, for which more than one subtype comprised nearly one-half of *pol*, we created trees with that CRF and both parent subtypes. Although CRF01_AE is itself a CRF, it is also the parent for several other CRFs. We used R packages including ape and phytools to identify the most recent common ancestor (MRCA) of the CRF and the sequence within the parent subtype with the shortest patristic distance to the MRCA. Overall 48 distinct sequences from 55 trees were identified of which 32 were not already included in our set of 684 representative sequences, yielding a final total of 716 GSPS sequences (GSPS.Set.txt, Data Citation 1).

Table 2 (available online only) describes the complete set of 6,034 one-person HIV-1 group M *pol* sequences and the 716 GSPS sequences. For each subtype/CRF, Table 2 (available online only) contains the number of sequences, the average pairwise distance (PWD) using the TN93 substitution model, and the number of sequences per country. It also describes the number of sequences per country in the GSPS panel and the overall ADCL for each subtype/CRF using the 716 GSPS sequences. Table 3 (available online only) lists the mean and maximum PWDs within each subtype/CRF for both the set of complete *pol* sequences and the GSPS subset. The data in the table indicates that the mean and maximum PWDs in the complete dataset and in the GSPS subset were very similar.

### Code availability

Scripts used for the present reference datasets generation are available from the site https://github.com/hivdb/Gene_to_Sequences

## Data Records

All reference datasets are available from Dryad Digital Repository (Data Citation 1). The complete set of 6,034 one-per-person, subtype-assigned HIV-1 group M complete *pol* sequences is available in a tab-delimited file containing the aligned *pol* sequence, GenBank accession number, GenBank author list, GenBank submission title, PubMed ID, country, sample year, and assigned subtype/CRF (Complete.Set.txt). The GSPS reference set of 716 sequences is also available in a tab-delimited file containing the GenBank accession number, country, sample year, PubMed ID, and assigned subtype/CRF (GSPS.Set.txt). Each of the 716 GSPS sequences is also included in a Fasta format (GSPS.Fasta.txt). The relationship between k and ADCL for each of the 155 subtype/CRF/country combinations with three or more sequences is listed in a tab-delimited file (ADCL.txt).

**Figure 2:**
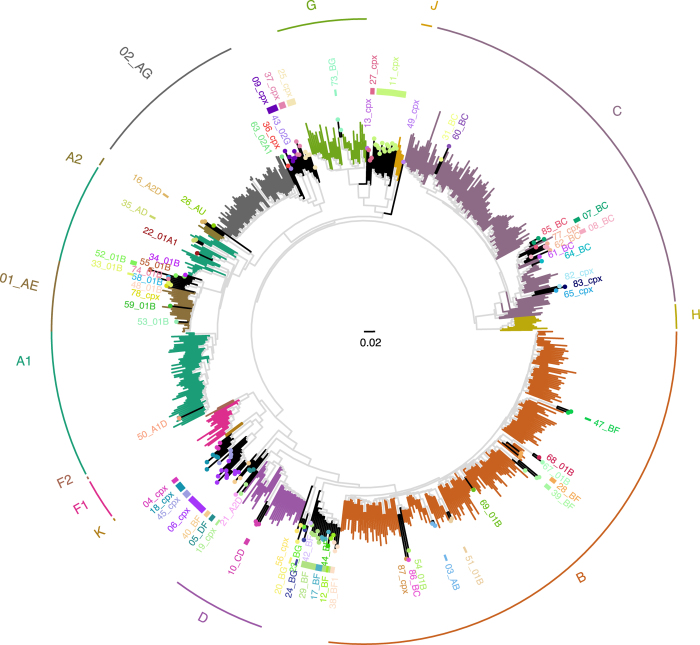
Phylogenetic tree of 716 Geographically-Stratified *Pol* Subtype/CRF (GSPS) reference sequences representing 11 pure subtypes and 70 CRFs. Branches of GSPS sequences belonging to the pure subtypes A1, A2, B, C, D, F1, F2, G, H, J, and K and the highly prevalent CRFs, CRF01_AE and CRF02_AG are color-coded and their clades are indicated in the outer ring. GSPS sequences belonging to the remaining 68 CRFs are indicated by black branches with color-coded circles on the branch tips. The tree was constructed using neighbour joining with branch length optimized by maximum likelihood method with GTR evolution model using the R package phangorn and then rooted at the mid-point. The tree was illustrated using the R package ggtree.

**Figure 3:**
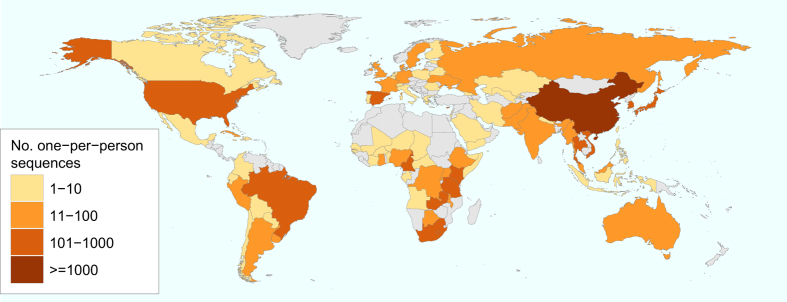
Number of sequences by country in the complete set of 6,034 one-per-person complete HIV-1 group M *pol* sequences. The large number of sequences from China is consistent with the frequent sequencing of complete genome and complete *pol* sequences in research and public health laboratories in this country.

**Figure 4:**
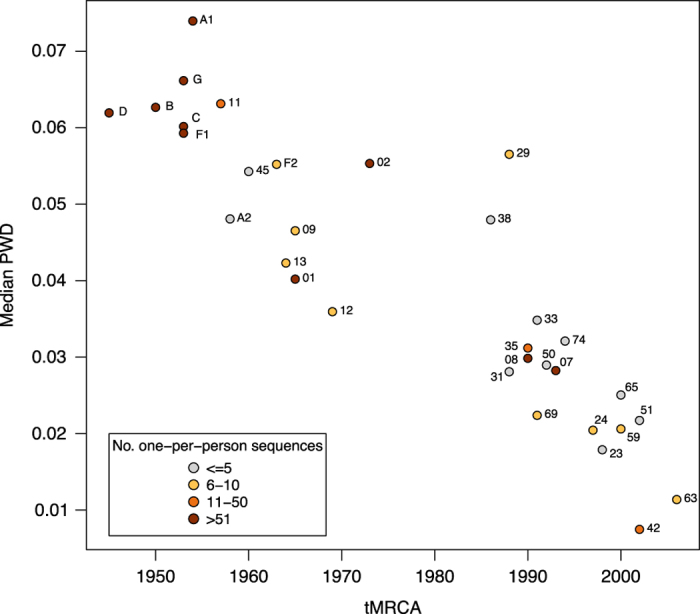
Correlation between the estimated year of the most recent common ancestor (MRCA) and the intra-subtype/CRF diversity in the complete set of 6,034 one-per-person complete HIV-1 group M *pol* sequences. Median PWD: median of the intra-subtype/CRF pairwise distances using TN93 substitution model. Each point indicates a subtype or CRF with CRFs labelled by their number alone. Points have been manually jittered to minimize overlap. Year of the MRCA was obtained from the references listed in Table 4.

A phylogenetic tree containing the 716 GSPS sequences is provided in Newick tree format (GSPS.tre) and in pdf format (Figure 2). Phylogenetic trees for each subtype/CRF are also provided in pdf format (GSPS.subtype.tre.pdf.zip). These trees were constructed using neighbour joining with branch length optimized by maximum likelihood method with GTR evolution model using the R package phangorn and then rooted at the mid-point. Figure 3 contains a map in which countries are color-coded according to number of complete *pol* sequences available. For those subtype/CRFs with published time to MRCA (tMRCA), Figure 4 (which is described in the Technical Validation section) plots the tMRCA year vs. the median intra-subtype/CRF PWD in the complete set of 6,034 sequences.

## Technical Validation

We performed three analyses to assess the how well the set of 716 GSPS sequences represent the diversity of published *pol* sequences. We also assess the potential usefulness of this set of sequences for subtyping. First, we determined whether the diversity within each subtype/CRF correlated with the age of the subtype/CRF as would be expected by viruses diverging from a common ancestor. Second, we compared the centrality of the GSPS sequences and NCBI Viral Genotyping Program reference sequences^8^ within the complete *pol* data set. We also determined whether the subtype/CRF of the closest GSPS sequence was the same as the subtype/CRF of the closest NCBI sequence. Third, we compared the genetic distance of the first 1,300 bps of the GSPS sequences to nearly 7,000 1,300 bp *pol* sequences for which the COMET and Rega subtyping programs assigned the same (i.e., consensus) subtype.

### Time to the most recent common ancestor (tMRCA) analysis

A strong correlation is expected between the diversity of sequences and tMRCA for each lineage as less divergence has taken place within lineages that originated more recently. To determine whether the diversity within each subtype/CRF in the 6,034 sequences from which the GSPS set was derived correlated with a published age of the subtype/CRF, we reviewed published papers that used molecular clock methods to estimate subtype and CRF tMRCAs. For those subtype/CRFs with published tMRCAs, we plotted the tMRCA year vs. the median intra-subtype/CRF PWD in the complete set of 6,034 sequences (Figure 4). Although the proportion of subtype/CRF sequences in the complete set is not representative of global epidemiology and the bias might have influenced the diversity of subtype/CRF in this set, there was a strong correlation between the published tMRCAs and the diversity of sequences in the complete set (Pearson’s correlation coefficient; r^2^=0.78; p<0.001). Table 4 lists the published papers with the tMRCA estimates used in this analysis.

### NCBI reference set comparison

To evaluate the centrality of the GSPS reference sequence set for distance-based subtyping, we compared the genetic distance of the GSPS sequences to the non-GSPS sequences in the complete *pol* data set with the genetic distances of the NCBI Viral Genotyping Program reference sequences^8^ to the non-NCBI reference sequences in the complete *pol* data set.

we compared the centrality of the 716 GSPS sequences with the 316 subtype reference sequences used by the NCBI Viral Genotyping Tool^8^. For this comparison, we used 5,185 test sequences–the complete set of 6,034 sequences excluding those sequences that were in either the GSPS or NCBI reference set. We then identified the subtype/CRF of the sequences in GSPS set and in the NCBI set with the fewest nucleotide differences from each test sequence.

For 96.0% of the test sequences, the subtype of the closest GSPS and NCBI reference sequences were the same. The median distance to the closest GSPS and NCBI reference sequences were 2.9% (IQR: 2.1–3.7%) and 4.2% (IQR: 2.9–5.0%), respectively (Wilcoxon rank-sum test: p<0.001). Thus, the GSPS set performed better at minimizing the distance to the test sequences. The increased centrality of the GSPS reference set may reflect the larger number of references in this set and/or the greater representativeness of its sequences.

Of the 4.0% (n=208) of sequences for which the subtype/CRF of the closest matching GSPS and NCBI sequences were not identical, most were not true discordances. For 126 sequences, the GSPS and NCBI subtypes were the same for the *pol* gene (i.e., the NCBI subtype was one of the eight subtype/CRFs that were reclassified by us because they did not contain *pol* recombination points). For 21 sequences, the GSPS subtype/CRF was one of the most recent CRFs that was not yet included in the NCBI set.

The subtype/CRF classifications of the remaining 61 sequences (1.2% of 5,185) with discordant classifications are shown in Table 5. Each of these sequences was closest to a CRF in the NCBI Reference Set but closest to a pure subtype in the GSPS set. For 26 of these sequences, the NCBI classification was likely to be incorrect because the test sequences were from Sub-Saharan Africa but their closest reference sequence was either CRF08_BC that has been reported primarily in China^23^ and CRF31_BC that has been reported primarily in Brazil^24^. The fact that NCBI CRF reference sequences were the closest sequence to likely pure subtype test sequences, suggests that the NCBI reference set may not have a sufficient number of representative pure subtype sequences. For the remaining 35 sequences, it was not possible to use isolate data to help infer the likely subtype/CRF.

### Genetic distance to 1,300 bp sequences with a consensus COMET/Rega subtype

To evaluate the potential utility of the GSPS reference sequence set for distance-based subtyping for partial *pol* sequences, we downloaded all one-per-patient HIV-1 group M sequences (n=50,335) encompassing the first 1300 bp of *pol* from LANL. We excluded the 6,034 complete *pol* sequences from the download because they were used to derive our GSPS set. We then randomly selected 1500 subtype B and 5500 non-subtype B sequences and aligned the first 1,300 bp of each of these sequences. For 6,115 (87.4%) of sequences, COMET and Rega assigned the same subtype/CRF. For the remaining 885 sequences, either COMET or Rega were discordant or reported that the sequence might have been a URF.

For 6,088 (99.6%) of the 6,115 sequences, the subtype/CRF of the closest sequence in the GSPS sequence set agreed with the consensus subtype/CRF assigned by COMET and Rega. The subtype/CRF classifications of the remaining 27 (0.4%) sequences with a discordance are shown in Table 6. The extraordinarily high concordance between the subtype/CRF of the closest GSPS sequence and the consensus subtype/CRF assigned by COMET/Rega likely reflects the fact that the test dataset contained only established subtypes and CRFs and did not contain URFs. We have not been able to identify any explanation for the few discordances listed in Table 6.

## Usage Notes

The set of quality-controlled, one-per-person 6,034 complete *pol* sequences annotated by country and sample year and assigned a subtype/CRF will be useful for HIV-1 molecular epidemiology studies that use *pol* sequence data. The set of 716 GSPS sequences will be useful for HIV-1 *pol* subtyping programs.

The software pipeline used to generate this dataset will be useful to identify, retrieve, align, and organize the annotations for newly submitted HIV-1 *pol* sequences. With different parameters, the software can also be used to identify, retrieve, and organize the annotations for other HIV-1 and virus genes. GB_to_BLASTDB.pl creates a local GenBank database and facilitates the rapid retrieval of GenBank annotations. Gene_to_Sequenes.pl accepts any gene protein sequence and generates an alignment of the gene-coding nucleotide sequences with GenBank annotations. It provides an option for the method of GenBank search, TBLASTN or TFASTX.

## Additional information

**How to cite this article**: Soo-Yon Rhee & Robert W. Shafer. Geographically-stratified HIV-1 group M pol subtype and circulating recombinant form sequences. *Sci. Data* 5:180148 doi: 10.1038/sdata.2018.148 (2018).

**Publisher’s note**: Springer Nature remains neutral with regard to jurisdictional claims in published maps and institutional affiliations.

## Supplementary Material



## Figures and Tables

**Table 1:** Proportions of each HIV-1 group M subtype/CRF in the set of 6,763 one-per-person complete *pol* sequences in GenBank compared with the reported global distribution of each subtype/CRF.

**Subtype/CRF**	**Global Distribution, %**^a^	**Complete** ***pol*** **Sequences, % (n=6,763)**^b^
C	48	18.0
A	12	4.6
B	11	32.2
CRF02_AG	8	1.9
CRF01_AE	5	17.9
G	5	1.1
D	2	1.3
F+H+J+K	1	1.4
Other CRFs	4	11.2
URFs	4	10.5

^a^Global distribution of HIV-1 obtained from the WHO-UNAIDS Network for HIV Isolation and Characterisation^22^. The data was collected between 2000 and 2007 from researchers and literature review.

^b^Proportion of complete *pol* sequences in GenBank (one per person) as of June 15, 2017.

**Table 2:** List of the Subtype/CRFs, Countries, and Intra-Subtype/CRF Divergence of the Complete Set of 6,034 Individuated *Pol* Sequences with Subtype Assignments and the Subset of 716 Representative Geographically-Stratified *Pol* Subtype (GSPS) Sequences.

Subtype / CRF	Complete Set (n=6,034)					GSPS (n=716)		Overall ADCL
	# Seqs	Mean PWD	#Countries	Countries (# Sequences per country)	Ancestor CRFs Identified	#Seqs	Countries (# Sequences per country)	
A1	306	0.071	24	KE(84), CY(32), TZ(30), UG(24), UA(21), CM(19), PK(19), RU(19), RW(11), UZ(8), IN(6), KZ(6), ZA(5), CD(4), SE(4), ES(3), SN(3), NG(2), AU(1), BY(1), GE(1), IT(1), SO(1), US(1)	CRF11_cpx, CRF22_01A1, CRF35_AD, CRF50_A1D	78	KE(20), TZ(8), UG(8), CM(6), CD(4), RW(4), SE(4), CY(3), ZA(3), NG(2), PK(2), SN(2), AU(1), BY(1), ES(1), GE(1), IN(1), IT(1), KZ(1), RU(1), SO(1), UA(1), US(1), UZ(1)	0.035
A2	4	0.050	3	CD(2), CM(1), CY(1)	CRF16_A2D, CRF21_A2D	4	CD(2), CM(1), CY(1)	NA
B	2175	0.062	42	US(751), JP(389), CN(261), BR(229), KR(112), CY(83), ES(58), AU(57), DE(36), TH(32), DK(17), AR(14), FR(13), PE(13), GB(10), JM(8), SE(8), RU(7), CA(6), CO(6), ZA(6), CU(5), HT(5), TT(5), UY(5), DO(4), GE(3), HK(3), MM(3), NL(3), PH(3), PY(3), UA(3), YE(3), CH(2), EC(2), TW(2), BO(1), GA(1), IN(1), MX(1), PL(1)	CRF03_AB, CRF07_BC, CRF20_BG, CRF23_BG, CRF24_BG, CRF28_BF, CRF38_BF1, CRF39_BF, CRF42_BF, CRF51_01B, CRF54_01B, CRF67_01B, CRF68_01B, CRF69_01B, CRF87_cpx	176	BR(22), ES(14), AR(13), PE(13), DE(12), JM(8), CO(6), DK(6), RU(6), CY(5), HT(5), SE(5), TH(5), CU(4), DO(4), ZA(4), CN(3), GE(3), JP(3), PY(3), TT(3), CH(2), EC(2), GB(2), PH(2), TW(2), US(2), UY(2), YE(2), AU(1), BO(1), CA(1), FR(1), GA(1), HK(1), IN(1), KR(1), MM(1), MX(1), NL(1), PL(1), UA(1)	0.031
C	1215	0.060	30	ZM(515), ZA(362), TZ(76), BW(54), CN(34), IN(33), MW(29), ET(26), BR(19), CY(12), SE(10), KE(9), NP(8), ES(5), US(5), CD(2), MM(2), NG(2), AO(1), AR(1), DE(1), DK(1), GE(1), IL(1), JP(1), PK(1), SN(1), SO(1), UY(1), YE(1)	CRF07_BC, CRF08_BC, CRF31_BD, CRF60_BC, CRF61_BC, CRF62_BC, CRF64_BC, CRF65_cpx, CRF77_cpx, CRF82_cpx, CRF83_cpx, CRF85_BC, CRF86_BC	126	MW(15), ET(14), SE(10), TZ(10), ZA(9), KE(8), NP(8), CN(7), BW(5), US(5), CY(4), ZM(4), BR(3), ES(3), IN(3), CD(2), MM(2), NG(2), AO(1), AR(1), DE(1), DK(1), GE(1), IL(1), JP(1), PK(1), SN(1), SO(1), UY(1), YE(1)	0.039
D	86	0.063	13	UG(45), KE(8), TZ(8), CD(7), CM(5), BR(3), TD(2), YE(2), ZA(2), CY(1), DK(1), KR(1), US(1)	CRF10_CD, CRF19_cpx, CRF21_A2D	33	KE(8), UG(7), CD(4), TZ(3), CM(2), YE(2), BR(1), CY(1), DK(1), KR(1), TD(1), US(1), ZA(1)	0.037
F1	66	0.055	10	BR(30), ES(22), AO(3), CD(2), FR(2), JP(2), RO(2), AR(1), FI(1), RU(1)	CRF12_BF, CRF17_BF, CRF29_BF, CRF38_BF1, CRF44_BF	16	BR(3), AO(2), CD(2), FR(2), JP(2), AR(1), ES(1), FI(1), RO(1), RU(1)	0.025
F2	9	0.054	1	CM(9)		2	CM(2)	0.039
G	77	0.063	13	NG(26), ES(19), CM(15), CD(3), CU(3), PT(3), KE(2), CN(1), GH(1), GW(1), IT(1), SE(1), ZA(1)	CRF20_BG, CRF23_BG, CRF24_BG, CRF43_02G, CRF73_BG	32	CM(10), NG(6), CD(3), ES(3), KE(2), CN(1), CU(1), GH(1), GW(1), IT(1), PT(1), SE(1), ZA(1)	0.033
H	10	0.061	5	CD(4), BE(2), CF(2), GB(1), US(1)		10	CD(4), BE(2), CF(2), GB(1), US(1)	NA
J	6	0.058	3	CD(3), SE(2), AO(1)		5	CD(3), AO(1), SE(1)	0.023
K	2	0.046	2	CD(1), CM(1)		2	CD(1), CM(1)	NA
01_AE	1210	0.040	15	CN(583), VN(384), TH(216), US(6), JP(4), CF(3), MM(3), SE(3), TW(2), AF(1), CM(1), HK(1), ID(1), IR(1), PH(1)	CRF33_01B, CRF34_01B CRF52_01B, CRF53_01B	24	TH(4), CF(3), SE(3), MM(2), TW(2), AF(1), CM(1), CN(1), HK(1), ID(1), IR(1), JP(1), PH(1), US(1), VN(1)	0.022
02_AG	130	0.055	20	CM(50), NG(23), GH(10), CY(8), PK(6), GW(5), FR(4), SE(4), SN(3), US(3), EC(2), ES(2), KR(2), RU(2), AO(1), DE(1), EE(1), MX(1), NE(1), TH(1)	CRF43_02G	58	NG(18), GH(5), GW(5), SE(4), CM(3), CY(3), SN(3), US(3), ES(2), KR(2), AO(1), DE(1), EC(1), EE(1), FR(1), MX(1), NE(1), PK(1), RU(1), TH(1)	0.037
03_AB	3	0.011	2	RU(2), BY(1)		2	BY(1), RU(1)	0.010
04_cpx	8	0.047	2	GR(6), CY(2)		3	GR(2), CY(1)	0.036
05_DF	3	0.047	3	BE(1), CD(1), ES(1)		3	BE(1), CD(1), ES(1)	
06_cpx	15	0.049	10	EE(4), KR(2), ML(2), BF(1), BJ(1), CD(1), GH(1), NG(1), RU(1), SN(1)		10	BF(1), BJ(1), CD(1), EE(1), GH(1), KR(1), ML(1), NG(1), RU(1), SN(1)	0.025
07_BC	300	0.028	3	CN(295), TW(4), MM(1)		3	CN(1), MM(1), TW(1)	0.019
08_BC	91	0.030	2	CN(89), MM(2)		3	MM(2), CN(1)	0.017
09_cpx	6	0.045	4	CI(2), SN(2), GH(1), US(1)		6	CI(2), SN(2), GH(1), US(1)	NA
10_CD	3	0.050	1	TZ(3)		3	TZ(3)	NA
11_cpx	23	0.063	6	CM(17), FR(2), CY(1), GR(1), NG(1), US(1)		15	CM(9), FR(2), CY(1), GR(1), NG(1), US(1)	0.038
12_BF	6	0.037	2	AR(4), UY(2)		2	AR(1), UY(1)	0.033
13_cpx	8	0.042	1	CM(8)		1	CM(1)	0.037
16_A2D	2	0.054	2	KE(1), KR(1)		2	KE(1), KR(1)	NA
17_BF	5	0.036	4	AR(2), BO(1), PE(1), PY(1)		4	AR(1), BO(1), PE(1), PY(1)	0.030
18_cpx	7	0.055	2	CM(4), CU(3)		5	CM(4), CU(1)	0.030
19_cpx	6	0.040	2	CU(4), ES(2)		2	CU(1), ES(1)	0.027
20_BG	4	0.023	2	CU(3), ES(1)		2	CU(1), ES(1)	0.012
21_A2D	3	0.042	1	KE(3)		1	KE(1)	0.040
22_01A1	1		1	CM(1)		1	CM(1)	NA
23_BG	2	0.018	1	CU(2)		1	CU(1)	0.018
24_BG	8	0.021	2	CU(7), ES(1)		2	CU(1), ES(1)	0.016
25_cpx	6	0.046	3	CM(3), SA(2), CD(1)		5	CM(3), CD(1), SA(1)	0.030
26_AU	1		1	CD(1)		1	CD(1)	NA
27_cpx	3	0.061	1	CD(3)		3	CD(3)	NA
28_BF	3	0.043	1	BR(3)		2	BR(2)	0.037
29_BF	7	0.056	1	BR(7)		7	BR(7)	NA
31_BC	2	0.028	1	BR(2)		1	BR(1)	0.028
33_01B	4	0.035	2	MY(3), ID(1)		2	ID(1), MY(1)	0.033
34_01B	3	0.008	1	TH(3)		1	TH(1)	0.006
35_AD	23	0.031	2	AF(14), IR(9)		2	AF(1), IR(1)	0.026
36_cpx	1		1	CM(1)		1	CM(1)	NA
37_cpx	4	0.055	2	CM(3), CY(1)		4	CM(3), CY(1)	NA
38_BF1	3	0.046	1	UY(3)		3	UY(3)	NA
39_BF	3	0.054	1	BR(3)		3	BR(3)	NA
40_BF	4	0.049	1	BR(4)		3	BR(3)	0.038
42_BF	21	0.008	1	LU(21)		1	LU(1)	0.004
43_02G	4	0.031	1	SA(4)		1	SA(1)	0.027
44_BF	1		1	CL(1)		1	CL(1)	NA
45_cpx	4	0.055	4	BR(1), CD(1), CM(1), GA(1)		4	BR(1), CD(1), CM(1), GA(1)	NA
47_BF	3	0.028	2	ES(2), BR(1)		2	BR(1), ES(1)	0.017
48_01B	3	0.024	1	MY(3)		1	MY(1)	0.023
49_cpx	3	0.041	1	GM(3)		1	GM(1)	0.040
50_A1D	4	0.030	1	GB(4)		1	GB(1)	0.025
51_01B	4	0.020	2	MY(2), SG(2)		2	MY(1), SG(1)	0.019
52_01B	3	0.032	2	TH(2), MY(1)		2	MY(1), TH(1)	0.025
53_01B	4	0.035	1	MY(4)		1	MY(1)	0.030
54_01B	2	0.027	1	MY(2)		1	MY(1)	0.027
55_01B	8	0.020	1	CN(8)		1	CN(1)	0.016
56_cpx	4	0.003	1	FR(4)		1	FR(1)	0.002
58_01B	6	0.026	1	MY(6)		1	MY(1)	0.023
59_01B	8	0.020	1	CN(8)		1	CN(1)	0.017
60_BC	3	0.021	1	IT(3)		1	IT(1)	0.020
61_BC	3	0.012	1	CN(3)		1	CN(1)	0.012
62_BC	3	0.018	1	CN(3)		1	CN(1)	0.018
63_02A1	10	0.011	1	RU(10)		1	RU(1)	0.009
64_BC	3	0.029	1	CN(3)		1	CN(1)	0.027
65_cpx	5	0.023	1	CN(5)		1	CN(1)	0.020
67_01B	2	0.016	1	CN(2)		1	CN(1)	0.016
68_01B	3	0.009	1	CN(3)		1	CN(1)	0.008
69_01B	7	0.021	1	JP(7)		1	JP(1)	0.013
73_BG	3	0.028	2	ES(2), DE(1)		2	DE(1), ES(1)	0.023
74_01B	3	0.032	1	MY(3)		1	MY(1)	0.029
77_cpx	4	0.018	1	MY(4)		1	MY(1)	0.017
78_cpx	3	0.031	1	CN(3)		1	CN(1)	0.029
82_cpx	6	0.017	1	MM(6)		1	MM(1)	0.011
83_cpx	11	0.012	1	MM(11)		1	MM(1)	0.009
85_BC	10	0.023	1	CN(10)		1	CN(1)	0.018
86_BC	3	0.033	1	CN(3)		1	CN(1)	0.031
87_cpx	3	0.028	1	CN(3)		1	CN(1)	0.026
Note: Pairwise distances (PWD) were calculated using TN93 substitution model.								
Abbreviations: PWD – pairwise distance; ADCL - average distance to the closest leaf; NA – not applicable.								

**Table 3:** Intra-Subtype/CRF Divergence of the Complete Set of 6,034 Individuated Pol Sequences and the Subset of 716 Representative Geographically-Stratified *Pol* Subtype (GSPS) Sequences According to Subtype/CRF.

Subtype/CRF	Complete Set (n=6,036)				GSPS (n=718)				Overall ADCL
	# Sequences	Mean PWD	Min PWD	Max PWD	# Sequences	Mean PWD	Min PWD	Max PWD	
A1	306	0.071	0.000	0.109	78	0.071	0.007	0.103	0.035
A2	4	0.050	0.046	0.057	4	0.050	0.046	0.057	NA
B	2175	0.062	0.000	0.102	176	0.065	0.009	0.100	0.031
C	1215	0.060	0.000	0.129	126	0.068	0.017	0.129	0.039
D	86	0.063	0.001	0.099	33	0.068	0.029	0.099	0.037
F1	66	0.055	0.002	0.086	16	0.063	0.032	0.083	0.025
F2	9	0.054	0.015	0.076	2	0.055	0.055	0.055	0.039
G	77	0.063	0.004	0.094	32	0.063	0.018	0.088	0.033
H	10	0.061	0.043	0.083	10	0.061	0.043	0.083	NA
J	6	0.058	0.023	0.071	5	0.061	0.050	0.071	0.023
K	2	0.046	0.046	0.046	2	0.046	0.046	0.046	NA
01_AE	1210	0.040	0.000	0.072	24	0.041	0.002	0.067	0.022
02_AG	130	0.055	0.002	0.086	58	0.056	0.005	0.080	0.037
03_AB	3	0.011	0.010	0.012	2	0.010	0.010	0.010	0.010
04_cpx	8	0.047	0.025	0.060	3	0.039	0.037	0.040	0.036
05_DF	3	0.047	0.035	0.055	3	0.047	0.035	0.055	NA
06_cpx	15	0.049	0.017	0.070	10	0.050	0.017	0.069	0.025
07_BC	300	0.028	0.000	0.053	3	0.025	0.016	0.034	0.019
08_BC	91	0.030	0.001	0.053	3	0.037	0.032	0.046	0.017
09_cpx	6	0.045	0.037	0.058	6	0.045	0.037	0.058	NA
10_CD	3	0.050	0.048	0.052	3	0.050	0.048	0.052	NA
11_cpx	23	0.063	0.006	0.085	15	0.065	0.050	0.085	0.038
12_BF	6	0.037	0.029	0.044	2	0.029	0.029	0.029	0.033
13_cpx	8	0.042	0.030	0.055	1	NA	NA	NA	0.037
16_A2D	2	0.054	0.054	0.054	2	0.054	0.054	0.054	NA
17_BF	5	0.036	0.029	0.041	4	0.036	0.029	0.041	0.030
18_cpx	7	0.055	0.028	0.074	5	0.058	0.046	0.074	0.030
19_cpx	6	0.040	0.021	0.056	2	0.051	0.051	0.051	0.027
20_BG	4	0.023	0.011	0.035	2	0.035	0.035	0.035	0.012
21_A2D	3	0.042	0.040	0.045	1	NA	NA	NA	0.040
22_01A1	1	NA	NA	NA	1	NA	NA	NA	NA
23_BG	2	0.018	0.018	0.018	1	NA	NA	NA	0.018
24_BG	8	0.021	0.009	0.034	2	0.022	0.022	0.022	0.016
25_cpx	6	0.046	0.030	0.054	5	0.049	0.040	0.054	0.030
26_AU	1	NA	NA	NA	1	NA	NA	NA	NA
27_cpx	3	0.061	0.056	0.068	3	0.061	0.056	0.068	NA
28_BF	3	0.043	0.037	0.050	2	0.043	0.043	0.043	0.037
29_BF	7	0.056	0.046	0.066	7	0.056	0.046	0.066	NA
31_BC	2	0.028	0.028	0.028	1	NA	NA	NA	0.028
33_01B	4	0.035	0.032	0.040	2	0.038	0.038	0.038	0.033
34_01B	3	0.008	0.002	0.012	1	NA	NA	NA	0.006
35_AD	23	0.031	0.002	0.044	2	0.022	0.022	0.022	0.026
36_cpx	1	NA	NA	NA	1	NA	NA	NA	NA
37_cpx	4	0.055	0.035	0.063	4	0.055	0.035	0.063	NA
38_BF1	3	0.046	0.042	0.049	3	0.046	0.042	0.049	NA
39_BF	3	0.054	0.050	0.058	3	0.054	0.050	0.058	NA
40_BF	4	0.049	0.038	0.058	3	0.052	0.047	0.058	0.038
42_BF	21	0.008	0.001	0.019	1	NA	NA	NA	0.004
43_02G	4	0.031	0.025	0.041	1	NA	NA	NA	0.027
44_BF	1	NA	NA	NA	1	NA	NA	NA	NA
45_cpx	4	0.055	0.046	0.063	4	0.055	0.046	0.063	NA
47_BF	3	0.028	0.017	0.034	2	0.032	0.032	0.032	0.017
48_01B	3	0.024	0.021	0.025	1	NA	NA	NA	0.023
49_cpx	3	0.041	0.037	0.045	1	NA	NA	NA	0.040
50_A1D	4	0.030	0.022	0.040	1	NA	NA	NA	0.025
51_01B	4	0.020	0.015	0.024	2	0.020	0.020	0.020	0.019
52_01B	3	0.032	0.025	0.038	2	0.033	0.033	0.033	0.025
53_01B	4	0.035	0.022	0.049	1	NA	NA	NA	0.030
54_01B	2	0.027	0.027	0.027	1	NA	NA	NA	0.027
55_01B	8	0.020	0.002	0.030	1	NA	NA	NA	0.016
56_cpx	4	0.003	0.002	0.005	1	NA	NA	NA	0.002
58_01B	6	0.026	0.014	0.036	1	NA	NA	NA	0.023
59_01B	8	0.020	0.002	0.030	1	NA	NA	NA	0.017
60_BC	3	0.021	0.018	0.023	1	NA	NA	NA	0.020
61_BC	3	0.012	0.011	0.013	1	NA	NA	NA	0.012
62_BC	3	0.018	0.018	0.018	1	NA	NA	NA	0.018
63_02A1	10	0.011	0.007	0.014	1	NA	NA	NA	0.009
64_BC	3	0.029	0.021	0.034	1	NA	NA	NA	0.027
65_cpx	5	0.023	0.014	0.030	1	NA	NA	NA	0.020
67_01B	2	0.016	0.016	0.016	1	NA	NA	NA	0.016
68_01B	3	0.009	0.007	0.010	1	NA	NA	NA	0.008
69_01B	7	0.021	0.002	0.038	1	NA	NA	NA	0.013
73_BG	3	0.028	0.023	0.033	2	0.029	0.029	0.029	0.023
74_01B	3	0.032	0.027	0.037	1	NA	NA	NA	0.029
77_cpx	4	0.018	0.007	0.025	1	NA	NA	NA	0.017
78_cpx	3	0.031	0.026	0.035	1	NA	NA	NA	0.029
82_cpx	6	0.017	0.004	0.035	1	NA	NA	NA	0.011
83_cpx	11	0.012	0.000	0.024	1	NA	NA	NA	0.009
85_BC	10	0.023	0.015	0.030	1	NA	NA	NA	0.018
86_BC	3	0.033	0.027	0.037	1	NA	NA	NA	0.031
87_cpx	3	0.028	0.025	0.031	1	NA	NA	NA	0.026
A1	306	0.071	0.000	0.109	79	0.072	0.007	0.103	0.035
A2	4	0.050	0.046	0.057	4	0.050	0.046	0.057	NA
B	2175	0.062	0.000	0.102	176	0.065	0.009	0.100	0.031
C	1215	0.060	0.000	0.129	126	0.068	0.017	0.129	0.039
D	86	0.063	0.001	0.099	33	0.068	0.029	0.099	0.037
F1	66	0.055	0.002	0.086	16	0.063	0.032	0.083	0.025
F2	9	0.054	0.015	0.076	2	0.055	0.055	0.055	0.039
G	77	0.063	0.004	0.094	32	0.063	0.018	0.088	0.033
H	10	0.061	0.043	0.083	10	0.061	0.043	0.083	NA
J	6	0.058	0.023	0.071	5	0.061	0.050	0.071	0.023
K	2	0.046	0.046	0.046	2	0.046	0.046	0.046	NA
Note: Pairwise distances (PWD) were calculated using TN93 substitution model.									
Abbreviations: PWD – pairwise distance; ADCL - average distance to the closest leaf; NA – not applicable									

**Table 4:** Time to the most recent common ancestors (tMRCA) of HIV-1 group M subtypes and CRFs.

**Subtype/CRF**	**Reference**	**tMRCA**	**CI**^**1**^
A1	Wertheim, J. O. *et al*^25^	1946	1936–1956
A2	Wertheim, J. O. *et al*^25^	1952	1941–1936
B	Wertheim, J. O. *et al*^25^	1955	1946–1964
C	Wertheim, J. O. *et al*^25^	1939	1926–1951
D	Abecasis, A.B. *et al*^26^	1945	1935–1955
F1	Wertheim, J. O. *et al*^25^	1950	1940–1959
F2	Wertheim, J. O. *et al*^25^	1961	1954–1968
G	Tongo, M. *et al*^27^	1953	1939–1963
01_AE	Liao, H. *et al*^28^	1967	1963–1973
02_AG	Faria, N.R. *et al*^29^	1973	1972–1975
07_BC	Tee, K.K. *et al*^30^	1993	1991–1995
08_BC	Tee, K.K. *et al*^30^	1990	1988–1991
09_cpx	Delatorre, E. *et al*^31^	1966	
11_cpx	Delatorre, E. *et al*^31^	1957	
12_BF	Dilernia, D. A. *et al*^32^	1969	1946–1981
13_cpx	Delatorre, E. *et al*^31^	1965	
20_BG	Delatorre, E. *et al*^33^	1996	1994–1998
23_BG	Delatorre, E. *et al*^33^	1998	1996–2000
24_BG	Delatorre, E. *et al*^33^	1997	1996–2000
28_BF	Ristic, N. *et al*^34^	1988	1984–1993
29_BF	Ristic, N. *et al*^34^	1988	1984–1993
31_BC	Passaes, C.P. *et al*^24^	1988	1982–1992
33_01B	Tee, K.K. *et al*^35^	1992	1987–1997
35_AD	Eybpoosh, S. *et al*^36^	1991	
38_BF1	Bello, G. *et al*^37^	1986	1981–1990
42_BF	Struck, D. *et al*^38^	2002	2001–2003
45_cpx	Delatorre, E. *et al*^39^	1965	
48_01B	Li, Y. *et al*^40^	2001	1998–2004
50_A1D	Foster, G. M. *et al*^41^	1992	1966–2007
51_01B	Ng, K. T. *et al*^42^	2000	1992–2006
59_01B	Zhang, W. *et al*^43^	2000	1994–2005
63_02A1	Shcherbakova, N. S. *et al*^44^	2006	2005–2007
65_cpx	Liu, Y. *et al*^45^	2000	1997–2003
69_01B	Hosaka, M. *et al*^46^	1993	1978–1999
74_01B	Cheong, H. T. *et al*^47^	1995	

**Table 5:** Test Sequences for which the Subtype/CRF of the Closest NCBI Reference Sequence Differed from the Subtype/CRF of the Closest Geographically-Stratified *Pol* Subtype/CRF (GSPS) Reference Sequence (n=61; 1.2% of 5,185 Test Sequences).

**NCBI**	**GSPS**	**# Sequences**	**Country/Region**^a^	**Notes**^b^
CRF01_AE	A1	5	Eastern Africa (3), South Africa (1), Cyprus (1)	CRF01_AE has been reported primarily in Southeast Asia and Central Africa. A1 is common in East Africa
CRF03_AB	B	1	US (1)	CRF03_AB has been reported primarily in Eastern Europe and Central Asia.
CRF08_BC	C	14	**Eastern Africa (n=7), Southern Africa (n=4)**, China (1), India (2)	CRF08_BC has been reported primarily in China.
CRF22_01A1	A1	9	Cameroon (9)	CRF22_01A1 has been reported primarily in Cameroon. Subtype A1 is also common in Cameroon
CRF28_BF	B	4	Brazil (4)	CRF28_BF has been reported primarily in Brazil. Subtype B is also common in Brazil.
CRF31_BC	C	15	**East Africa (n=11), Southern Africa (n= 4)**	CRF31_BC has been reported primarily in Brazil.
CRF51_01B	B	2	Japan (1), Philippine (1)	CRF51_01B has been reported primarily in Southeast Asia and Japan. Subtype B is common in Japan.
CRF64_BC	C	6	China (6)	CRF64_BC has been reported primarily China. Subtype C is also common in China.
CRF69_01B	B	5	Japan (5)	CRF69_01B has been reported primarily in Japan. Subtype B is common in Japan

^a^Sequences for which the closest NCBI sequence are unlikely to represent the correct subtype/CRF of the sequences are shown in bold.

^b^The information in this column was obtained from Los Alamos National Laboratories (LANL) HIV Sequence Database^1^.

**Table 6:** 1300 bp *pol* Test Sequences for which the Consensus Subtype/CRF assigned by COMET and Rega Differed from the Subtype/CRF of the Closest Geographically-Stratified *Pol* Subtype/CRF (GSPS) Reference Sequence (n=27; 0.4% of the 6,115 Test Sequences with a Consensus Subtype).

**Consensus Subtype/CRF**^a^	**GSPS**^b^	**# Sequences**	**Country**	**Notes**^c^
CRF02_AG	CRF36_cpx (1)	1	Cameroon (1)	CRF02_AG is common in Cameroon. CRF36_cpx has been reported primarily in Cameroon.
CRF12_BF	CRF17_BF (1), B (3)	4	Argentina (4)	CRF12_BF and CRF17_BF has been reported primarily in South America. Subtype B is also common in South America.
A1	CRF22_01A1 (3)	3	Cameroon (3)	Subtype A1 is common in Cameroon. CRF22_01A1 has been reported primarily in Cameroon.
A1	A2 (1)	1	Republic of the Congo (1)	Subtypes A1 and A2 are common in Republic of the Congo.
B	CRF17_BF(1), CRF38_BF1 (2)	3	Argentina (3)	Subtype B and CRF17_BF are common in South America. CRF38_BF1 has been reported primarily in Uruguay.
B	CRF28_BF (1)	1	Brazil (1)	Subtype B and CRF28_BF are common in Brazil.
C	CRF07_BC (1), CRF85_BC (3)	4	China (4)	Subtype C is common in China. CRF07_BC and CRF85_BC have been reported primarily in China.
D	B (3)	3	Republic of the Congo (1), South Africa (1), Spain (1)	Subtype D is generally only seen in Eastern and Central Africa.
G	CRF43_02G (1)	1	Nigeria (1)	CRF43_02G is primarily reported in Saudi Arabia. Subtype G has been reported commonly in Africa.
G	CRF73_BG (6)	6	Portugal (4), Spain (2)	Subtype G is common in Central Europe. CRF73_BG has been reported in Portugal and Spain.

^a^Subtype/CRF classifications of test sequences that were agreed by COMET and Rega subtyping programs.

^b^Subtypes/CRFs of the closest GSPS sequences of the test sequences that differed from their consensus subtype/CRF.

^c^The information in this column was obtained from Los Alamos National Laboratories (LANL) HIV Sequence Database^1^.
